# Long non-coding RNA XIST promotes osteoporosis by inhibiting the differentiation of bone marrow mesenchymal stem cell by sponging miR-29b-3p that suppresses nicotinamide N-methyltransferase

**DOI:** 10.1080/21655979.2021.1967711

**Published:** 2021-09-05

**Authors:** Jiang Yu, Min Xiao, Guohai Ren

**Affiliations:** a Department of Orthopedics Surgery, Affiliated Hospital of Jianghan University, Wuhan, China; b Department of Internal Schistosomiasis Ward, Wuhan Daishan Hospital, Wuhan, China

**Keywords:** XIST, BMSCS, MIR-29B-3P, NNMT, osteoporosis

## Abstract

Bone formation is important in the development of osteoporosis (OP). X–inactive specific transcript (XIST), a lncRNA, is involved in this process; however, mode of its action is not known. We compared the serum levels of XIST and miR-29b-3p among the patients with and without OP. In rat bone marrow mesenchymal stem cells (BMSCs), during osteogenic differentiation, XIST expression was detected first, followed by overexpression or suppression of miR-29b-3p and NNMT. Expression of osteogenic genes, ALP (electrochemical alkaline phosphatase) and RUNX2 (Runt-related transcription factor 2) were detected by RT-qPCR and western blots, and the calcium nodules in BMSCs were detected by staining. The relationships of XIST, miR-29b-3p, and NNMT were characterized by dual-luciferase reporter assay. Serum XIST was significantly upregulated in patients of OP. XIST downregulated the ALP and Runx2 levels and inhibited calcium nodules, whereas low expression of XIST reversed these events. MiR-29b-3p was inhibited by XIST sponge and lowered the levels of ALP, Runx2, and calcium nodules. NNMT was negatively regulated by miR-29b-3p, promoting the osteogenic differentiation of BMSCs. In conclusion, XIST is highly expressed in OP, and regulates NNMT by sponging miR-29b-3p to suppress the osteogenic differentiation of BMSCs.

## Introduction

Osteoporosis (OP), a metabolic disease, is characterized by the damaged bone structure and low bone mass, making them more likely to fracture [1], which affects the health and quality of life of the patients and shortens their life span [2]. Currently, there are about 200 million patients of OP globally, and this situation has seriously increased the financial and medical burden of the families and countries [3]. OP causes an imbalance between the bone formation by osteoclasts and bone resorption by osteoblasts [4]. Bone marrow mesenchymal stem cells (BMSCs) are considered to be ideal seed cells for cell regeneration therapy because of the low expression of immunogens on their surface and their ability to self-renew [5]. These cells participate in bone formation and osteogenic differentiation [6]. Therefore, improving the ability of BMSCs to undergo osteogenic differentiation is important in alleviating osteoporosis.

Long non-coding RNA (lncRNA) regulates gene expression at epigenetic, transcriptional, and post-transcriptional levels, but does not encode any protein [7,8]. Recently, lncRNAs are shown to be important in a variety of skeletal diseases, including OP, osteoarthritis, and osteosarcoma [9]. For example, knockdown of small nucleolar RNA host gene 5 (SNHG5), a lncRNA, inhibits the osteogenic differentiation of BMSCs and induces apoptosis [10]. Two lncRNAs, AK079370 and AK039312, suppress bone formation and osteoblast differentiation by inhibiting osteogenic transcription factors [11]. Another lncRNA, X–inactive specific transcript (XIST), is involved in many disorders, and is overexpressed in patients of OP, and inhibits the differentiation of BMSCs into osteoblasts [12]. In mouse model, XIST alleviates osteoporosis induced by accumulation of iron [13]. It is significantly enhanced in the serum of patients of osteoporosis and suppresses osteogenic differentiation of BMSCs [14]. In addition, in BMSCs XIST is shown to increase as the animals aged. Knockdown of XIST induces ALP activity and secretion of osteocalcin [15]. However, the underlying mechanism through which it promotes the development of OP remains unclear.

Nicotinamide N-methyltransferase (NNMT) is a metabolic enzyme involved in carbon metabolism and methylation, and can have either physiological or pathological role, based on its distribution in tissues [8]. In addition to its role in cancer, recent investigations have shown that NNMT is involved in the ceRNA network in BMSCs from ovariectomized (OVX) mice. However, its specific role in OP remains unknown.

In the present study, a competing endogenous RNA (ceRNA) network, constituted by XIST and NNMT during OP progression, is proposed. Our bioinformatics analyses identified the involvement of miR-29b-3p in the ceRNA network formed by XIST and NNMT. MiR-29b-3p is a microRNA (miRNA) involved in cell proliferation, differentiation, and apoptosis through conditioned mRNA in the metabolic activity of bone tissue cells in vivo [16]. It is expressed at low levels in the exosomes of BMSCs derived from patients of OP, and osteogenic differentiation was promoted by targeting the enzyme KDM5A [9]. Therefore, we suspect that the XIST/miR-29b-3p/NNM axis may be critically involved in OP. We aim to clarify the effect of the XIST/miR-29b-3p/NNM axis during OP pathogenesis. Our findings may open a new avenue for diagnosis and treatment of OP .

## Materials and methods

### Bioinformatics analysis

GSE35959, an mRNA microarray from GEO DataSet, which included OP and non-OP samples, was used to screen the differentially expressed genes (DEGs) with adjusted P < 0.05, and |logFC| ≥1.5. Protein–protein interactions of DEGs were analyzed using STRING. StarBase was used to predict the miRNAs binding to XIST and NNMT1. GSE91033 including OP samples and non-OP samples from GEO DataSets was a miRNA microarray to screen the differentially expressed miRNAs (DE-miRNAs) with P < 0.05, and |logFC| ≥1.5.

### Clinical samples

This clinical study was approved by the medical ethics committee, and informed consent was obtained from all participants. Twenty patients of OP and 20 with non-osteoporotic fractures, admitted to our hospital, were included in this study. All patients were fasting for 8 hours after admission, and then venous blood was collected using non-anticoagulant red blood cells. The venous blood was placed at 4°C for 30 min and then centrifuged at 4°C for 2,500 × g for 15 min. Supernatant serum was collected and stored at −20°C.

### Laboratory animals

Three-week-old female Sprague-Dawley (SD) rats were procured from Hunan Slac Jingda Laboratory Animal Co. Ltd. (China). All animal experiments were approved by the Animal Care and Use Committee of our Laboratory Animal Research Center. All animals were reared under specific pathogen-free conditions at 22°C, under 12 h light/12 h darkness cycle, with 50–55% humidity, and had free access to food and water.

### BMSCs differentiation

The bone marrow of rat tibia and femur [10,11] was flushed out with PBS, collected in DMEM supplemented with 1% HEPES, 1% penicillin, and 10% FBS (Gibco; Thermo Fisher Scientific, USA), and incubated at 37°C, at 5% CO_2_. Non-adherent cells were discarded by changing the culture medium every 2 days. After 5 days, the cells were subcultured with 0.25% trypsin. After 3–5 generations in culture, the cells were transferred to osteogenic medium to induce osteogenic differentiation of BMSCs. The osteogenic medium was composed of 10% FBS, 50 μg/mL ascorbic acid, 10 nmol/L dexamethasone, 10 mmol/L β-glycerol phosphate, 1% L-glucose, 1% penicillin-streptomycin, 1% HEPES, in high glucose-DMEM (DMEM-HG; Gibco).

### Cell transfection

XIST overexpression plasmid pcDNA-XIST and corresponding negative control pcDNA-NC, XIST interference plasmid sh-XIS #1, sh-XIS #2, sh-XIS #3, and corresponding negative control sh-NC, Antago mature rat miR-29b-3p, and corresponding negative control Antagomir-NC, NNMT interference plasmid sh-NNMT# 1, sh-NNMT# 2, sh-NNMT# 3, and corresponding negative control sh-NC were designed and synthesized by GenePharma (China). BMSCs were cultured for 24 h before transfection. pcDNA (1 μg/mL), shRNA (50 nM), or antago miRNA (100 nM) were transfected into BMSCs using Lipofectamine 2000 kit (Invitrogen, USA). Follow-up experiments were performed 48 h after transfection.

### RT-qPCR

miRNAs from plasma were isolated using Qiagen miRNeasy serum/plasma kit (Qiagen, Germany), and those from cells were purified using miRNA isolation kit (Ambion). These were reverse transcribed using Ncode miRNA first-strand cDNA synthesis kits (Invitrogen). MiR-29b-3p was reverse transcribed using miRNA-specific loop primers, and amplified with ABI7900 Fast Real-Time System (Applied Biosystems, USA) using Taqman Micrornas (Applied Biosystems). The SnRNA U6 was used as a standard reference marker.

Total mRNA was extracted from the blood and cells using Eastep® Super RNA Extract reagent Kit (Promega, USA), following to the manufacturer’s instructions, and cDNA was synthesized with a reverse transcription kit (Takara, Japan). Power SYBR Green PCR Master Mix (Promega) was used for real-time PCR on QuantStudio 6 Flex (Applied Biosystems). GAPDH served as an internal control. The primers used are listed in Table 1.

**Table 1.** The sequences of the PCR primers in this study

Primer	Sequences
**XIST**	Forward: 5’-GCTCTTCATTGTTCCTATCTGCC-3’
	Reverse: 5’-TGTGTAAGTAAGTCGATAGGAGT-3’
**NNMT**	Forward: 5’-GAATCAGGCTTCACCTCCAA-3’
	Reverse: 5’-CCCAGGAGATTATGAAACACC-3’
**ALP**	Forward: 5’-GACCTCCTCGGAAGACACTC-3’
	Reverse: 5’-TGAAGGGCTTCTTGTCTGTG-3’
**Runx2**	Forward: 5’-TCTTAGAACAAATTCTGCCCTTT-3’
	Reverse: 5’-TGCTTTGGTCTTGAAATCACA-3’
**miR-29b-3p**	Forward: 5’-CTGCTAGCACCATTTGAAA-3’
	Reverse: 5’-GTGCAGGGTCCGAGGT-3’
**GAPDH**	Forward: 5’-ACGGATTTGGTCGTATTGGG-3’
	Reverse: 5’-CCTGGAAGATGGTGATGGGATT-3’
**U6**	Forward: 5’-CTCGCTTCGGCAGCACA −3’
	Reverse: 5’-AACGCTTCACGAATTTGCGT −3’

### ARS staining

The cells were incubated in osteoblast medium for 14 days, and stained for the deposited minerals, if any. For this, the cells were fixed for 20 min at 25°C with paraformaldehyde, gently washed with PBS, and then stained with 1% ARS solution at 25°C for 10 min. After washing with PBS, images were captured under a light microscope.

### Western blot

BMSCs were lysed, and the protein concentration was measured by BCA method (Thermo Fisher Scientific) and adjusted by radioimmunoprecipitation (Beyotime, China). The proteins were separated by SDS-PAGE at 30 μg/lane concentration, and transferred to a PVDF membrane (Millipore, Bedford, MA, USA). After this, the membrane was soaked in 5% skimmed milk for 1 h at 25°C, and incubated overnight at 4°C with the antibodies against RUNX2 (ab23981, Abcam, UK), ALP (ab229126, Abcam), NNMT (ab119758, Abcam), and GAPDH (ab9485, Abcam). After washing with PBS, the membrane was incubated at 37°C for 1 h in appropriate secondary antibody. Finally, the relevant protein bands were detected using ECL kits (Santa Cruz, USA) and the expression profiles of different proteins were analyzed using Image J Software (Version 1.38; NIH, USA). GAPDH was used as an internal control.

### Dual-luciferase reporter assay

The XIST and NNMT 3’-UTR fragments containing miR-29b-3p binding sites were amplified by PCR, and the products were inserted into PGL3 controlled luciferase reporter vector (Promega), to prepare WT-PGL3-XIST or WT-PGL3-NNMT constructs. Mutation of the binding site of miR-29b-3p using Quik-Change™ Site-Directed Mutagenesis Kit (Stratagene, CA, USA), specific mutations were induced in the binding site of miR-29b-3p, to prepare MUT-PGL3-XIST or MUT-PGL3-NNMT constructs. Either agomiR-29b-3p or agomiR-NC was co-transfected into 293 T cells along with the constructed luciferase reporter vectors for 48 h. Luciferase activity in the recipient cells was quantified with dual-luciferase reporter system (Promega) using a photometer (Glomax; Promega). The firefly luciferase was normalized to Renilla luciferase.

## Statistical analyses

SPSS software (SPSS Statistics, USA) and GraphPad Prism 5.0 (GraphPad Software, USA) were used for statistical analyses and image editing. Data are presented as mean ± standard deviation. Differences between two groups were compared with Students’ t-test, and those between multiple groups were assessed with one-way analysis to compare the multiple groups. All representative experiments were repeated at least three times. Statistical significance was set at P < 0.05.

## Results

### XIST/miR-29b-3p/NNMT axis may play the key role in osteoporosis

**Figure 1.**
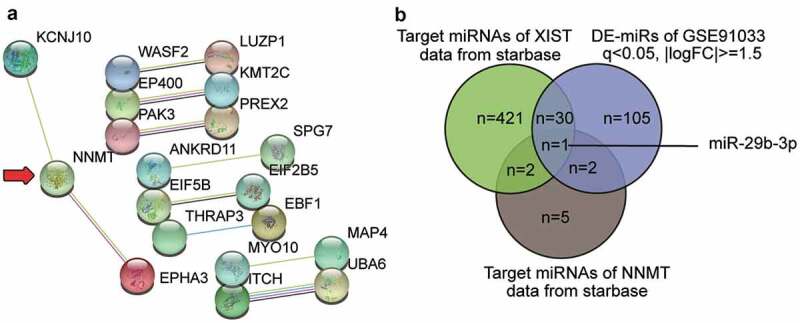
**XIST/miR-29b-3p/NNMT axis may participate in osteoporosis**.A. The protein-protein interaction analysis of the top 50 most significantly upregulated genes of GSE35959 data series (adjusted P < 0.05 and |logFC|≥1.5) by string algorithm. B. The intersection between the target miRNAs of XIST, the target miRNAs of NNMT, and the differentially expressed miRNAs in osteoporosis by analyzing GSE91033. The target prediction of XIST and NNMT was conducted using starbase algorithm. The DE-miRNAs of GSE91033 were selected at adjusted P < 0.05 and |logFC|≥1.5

To further understand the role of XIST in OP, we analyzed GSE35959 profiling and identified a total of 109 DEGs in elderly patients of OP, using the criteria of adjusted P < 0.05, and |logFC| ≥1.5 (Supplementary Table S1). We uploaded the 50 most significantly upregulated DEGs to the string for analysis of protein–protein interaction. The result shown in Figure 1a suggests that NNMT1 closely interacts with KCNJ10 (potassium voltage-gated channel subfamily J member 10) and EPHA3 (erythropoietin-producing hepatocellular carcinoma A3). As NNMT1 is significantly upregulated in OVX-induced osteoporosis [12], we further explored its role in osteoporosis. We also identified the potential miRNAs that link XIST and NNMT1 by intersecting the target miRNAs of XIST, the target miRNAs of NNMT1, and the DE-miRNAs of GSE91033. miR-29b-3p, which accelerates bone formation by downregulating the inhibitors of osteogenic differentiation, and the loss of which leads to frequent bone fractures [15], was also identified (Figure 1b).

### XIST expression in OP and during osteogenic differentiation

**Figure 2.**
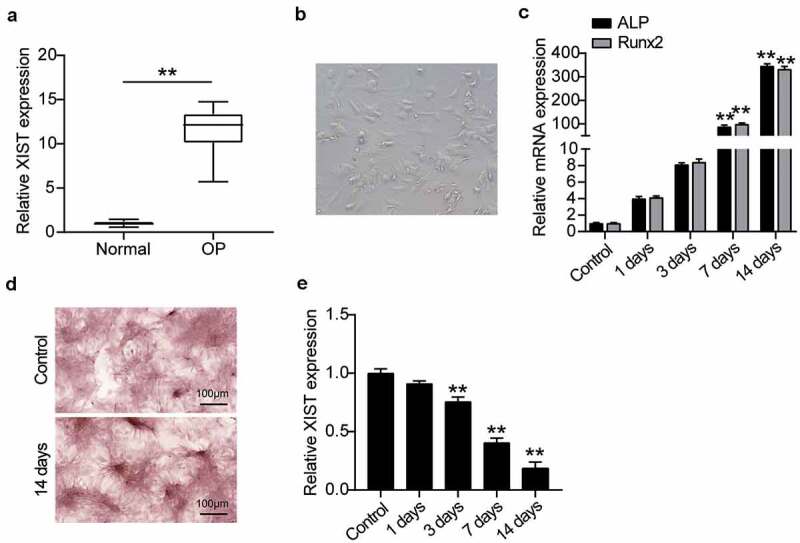
**XIST is highly expressed in OP and is low expressed during osteogenic differentiation**.(a) XIST expression in the serum of OP patients and non-OP patients. ** P < 0.001. (b) Phenotypic identification of bone marrow mesenchymal stem cells. The shape of BMSCs on the 4th day. (c) The expression levels of osteoblast marker genes ALP, RUNX2 on osteogenesis differentiation. (d) Following cultured in osteogenic induction medium for 14 days, B MSCs exhibited more mineralized nodules according to ARS staining, whereas the control group did not. (e) The expression of XIST during the time of osteogenesis differentiation

To understand the role of XIST in OP, first the level of XIST expression in the serum of patients with non-osteoporotic fractures was compared with that of patients of OP, on RT-qPCR. Results showed that the XIST expression was about 12 times higher in patients of OP over that of the non-OP patients (Figure 2a), which suggested that greater expression of XIST may be related to OP. We then studied the mechanism of action of XIST at the cellular level using BMSCs. For this, cell morphology was observed on the fourth day after the induction of osteogenic differentiation. Induced cells grew well and showed spindle growth (Figure 2b). Expression levels of key osteogenic genes ALP and RUNX2 were detected, suggesting that ALP and RUNX2 expression increased with osteogenic differentiation (Figure 2c). ARS staining showed the calcium deposition upon osteogenic differentiation after 14 days of induction (Figure 2d), suggesting the successful induction of BMSCs into osteogenic lineage. Monitoring the XIST expression level during osteogenic differentiation showed its downregulation as the differentiation progressed (Figure 2e).

** P < 0.001 vs. Control.

### XIST inhibited osteogenic differentiation of BMSCs

**Figure 3.**
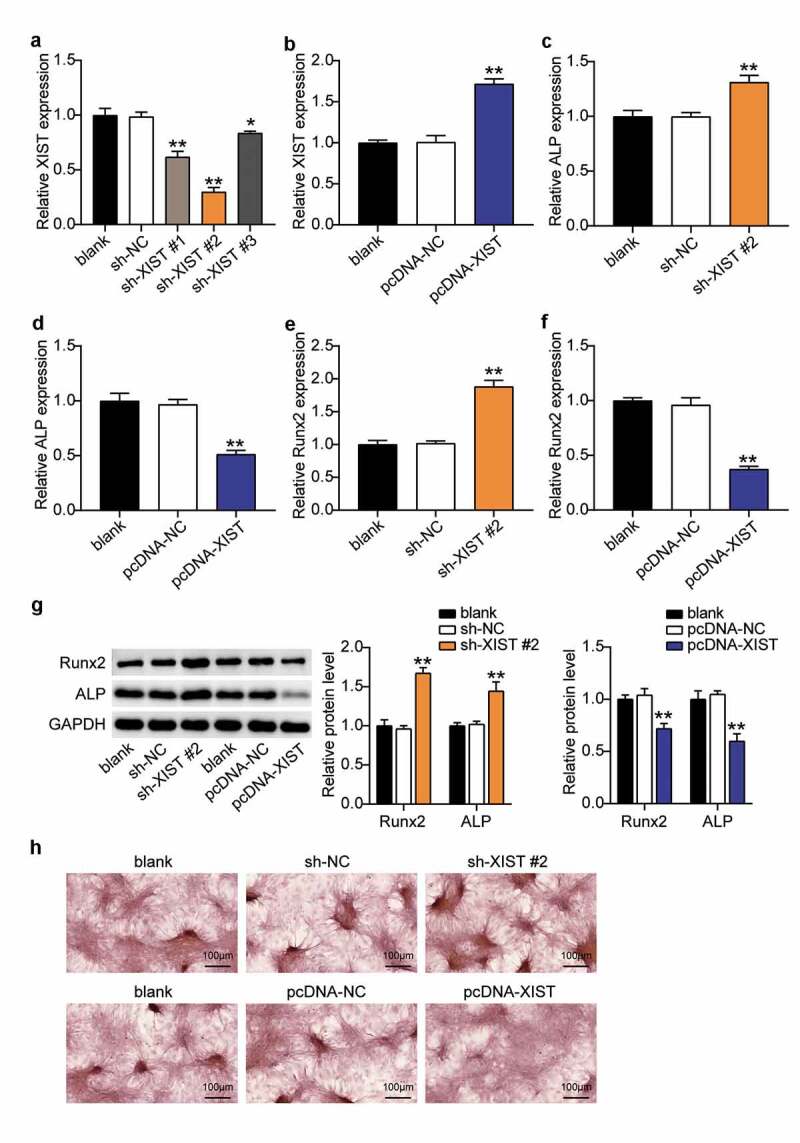
**Down-regulation of XIST promotes BMSCs osteogenic differentiation, and up-regulation of XIST inhibits BMSCs osteogenic differentiation**.(a) The expression of XIST in BMSCs after transfection of sh-XIST #1, sh-XIST 2# or sh-XIST 3#. (b) The expression of XIST in BMSCs after transfection of pcDNA-XIST. (c) The expression of ALP mRNA in BMSCs after transfection of sh-XIST 2#. (d) The expression of ALP mRNA in BMSCs after transfection of pcDNA-XIST. (e) The expression of RUNX2 mRNA in BMSCs after transfection of sh-XIST 2#. (f) The expression of RUNX2 mRNA in BMSCs after transfection of pcDNA-XIST. (g)The expression of RUNX2 and ALP protein in BMSCs after transfection of sh-XIST 2# or pcDNA-XIST. (h) Calcium deposition in BMSCs transfected with sh-XIST 2# or pcDNA-XIST was detected by ARS staining

To explore the role of XIST in the osteogenic differentiation of BMSC, the XIST knockdown plasmid construct was transfected into BMSCs, and the results revealed that XIST expression levels in the sh-XIST #1, sh-XIST 2#, and sh-XIST 3# groups were downregulated nearly 40%, 70%, and 20%, respectively, compared with the sh-NC group (Figure 3a), indicating that XIST was successfully knocked down in these cells. Cells from the sh-XIST 2# group was used for subsequent experiments. To establish the cell lines overexpressing XIST, BMSCs were transfected with pcDNA-XIST and pcDNA-NC. RT-qPCR analysis showed that the expression level of XIST in the pcDNA-XIST group increased by almost 1.7-fold over the levels seen in the pcDNA-NC group (Figure 3b). Expression of ALP increased by about 1.3 times, and that of RUNX2 rose by 1.8 times after XIST knockdown, whereas both these reduced by 50% and 70%, respectively, upon XIST overexpression (Figure 3c, D, E, F), suggesting the influence of XIST on ALP and RUNX. Western blots supported these observations; expression of ALP and RUNX2 proteins increased 1.4-fold and 1.7-fold after XIST interference, and decreased by 40% and 25% after XIST up-regulation (Figure 3g). Moreover, ARS staining showed that calcium nodules increased after transfecting sh-XIST 2# into BMSCs, and decreased after transfection with pcDNA-XIST (Figure 3h).

* P < 0.05, ** P < 0.001 vs. blank.

### XIST targets miR-29b-3p

**Figure 4.**
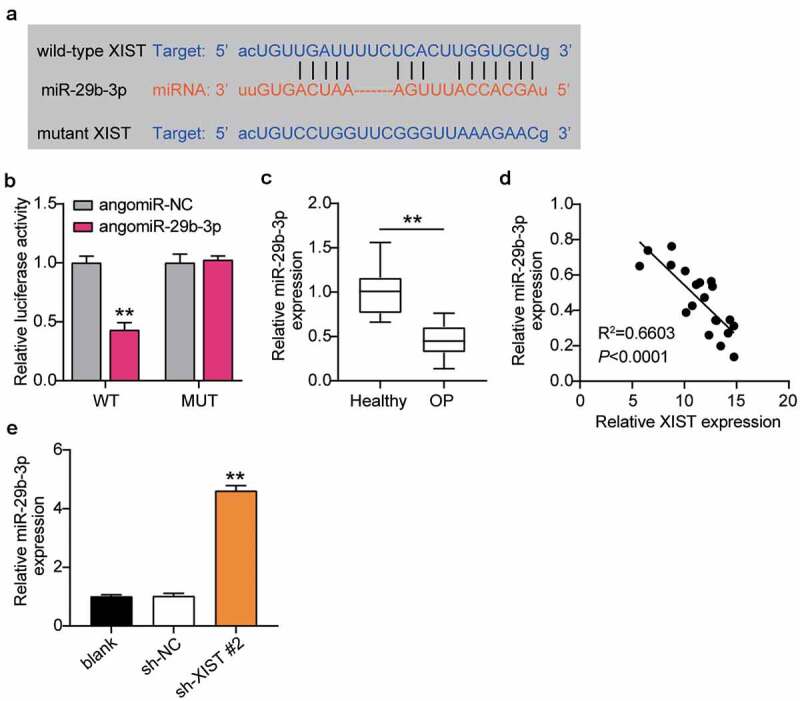
**XIST targets miR-29b-3p**.(a) Schematic diagram of predicted binding sites of XIST in 3’-UTR of miR-29b-3p. (b) Determination of the dual-luciferase activity of BMSCs transfected with XIST-MUT or XIST-WT and agomiR-NC or agomiR miR-29b-3p. ** P < 0.001 vs. agomiR-NC. (c) The expression of miR-29b-3p in the serum of OP patients and non-OP patients. ** P < 0.001. (d) Pearson analysis of correlation between miR-29b-3p and XIST in the serum of OP patients. (e) The expression of miR-29b-3p in BMSCs after transfection of sh-XIST 2#

As the ceRNA regulatory network is the main way of lncRNA regulating OP, we used starBase analysis to predict the targets of XIST. The starBase website shows that XIST and miR-29b-3p have target sites (Figure 4a). The relationship between XIST and miR-29b-3p was examined by dual-luciferase reporter assay, to find that the luciferase activity decreased after transfection of WT-XIST and agomir miR-29b-3p, but not after transfection of MUT-XIST and agomiR miR-29b-3p (Figure 4b). RT-qPCR detected miR-29b-3p expression in patients of OP, and found that miR-29b-3p expression was lower in patients of OP than in non-OP patients (Figure 4c). Pearson analysis suggested a negative correlation between the expression levels of XIST and miR-29b-3p in the serum of patients of OP (Figure 4d). RT-qPCR supported this observation, and showed that downregulation of XIST enhanced the level of miR-29b-3p expression by 4.5 times (Figure 4e). These observations together revealed that XIST targets and negatively regulates the expression of miR-29b-3p in OP.

** P < 0.001 vs. blank.

### Silencing XIST relieves the effect of miR-29b-3p inhibitor on osteogenic differentiation

**Figure 5.**
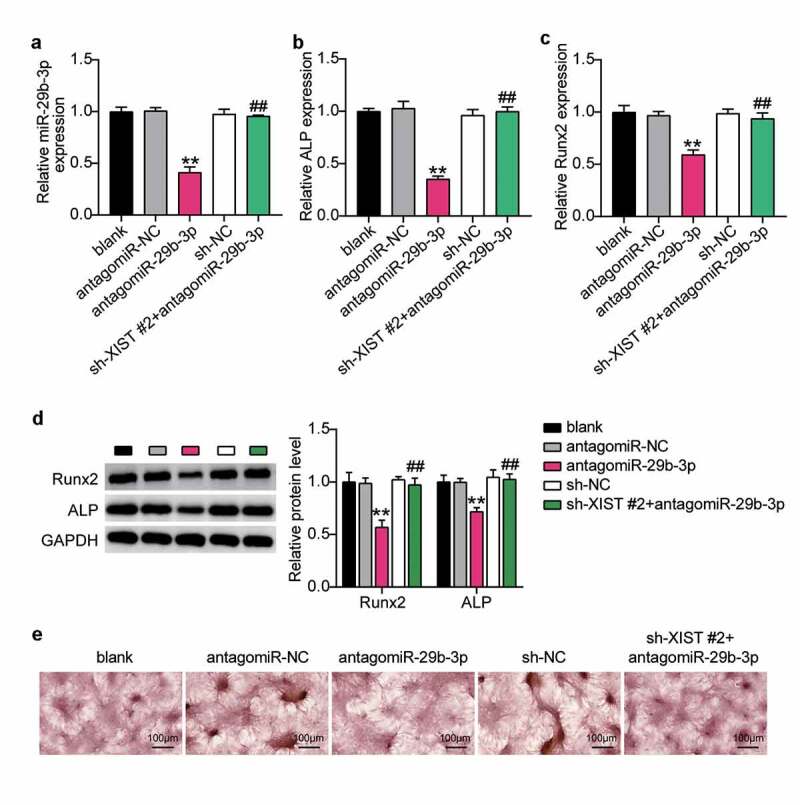
**XIST inhibits osteogenic differentiation by inhibiting miR-29b-3p**.(a) The expression of miR-29b-3p in BMSCs after transfection of antagomiR miR-29b-3p or sh-XIST 2#. (b) The expression of ALP mRNA in BMSCs after transfection of antagomiR miR-29b-3p or sh-XIST 2#. (c) The expression of RUNX2 mRNA in BMSCs after transfection of antagomiR miR-29b-3p or sh-XIST 2#. (d) The expression of RUNX2 and ALP protein in BMSCs after transfection of antagomiR miR-29b-3p or sh-XIST 2#. (e) Calcium deposition in BMSCs transfected with antagomiR miR-29b-3p or sh-XIST 2# was detected by ARS staining

To find out whether XIST regulates osteogenic differentiation through miR-29b-3p, we first transfected antago miR-29b-3p into BMSCs to find that the level of miR-29b-3p decreased by 60%, which was reversed by downregulation of XIST (Figure 5a). Expression levels of ALP and Runx2 decreased by 70% and 40%, after downregulation of miR-29b-3p. Interference with XIST did not increase these levels (Figure 5BC). Similarly, western blots showed that ALP and Runx2 decreased after miR-29b-3p deficiency, while co-transfection of sh-XIST with antago miR-29b-3p showed no significant change in these expression profiles (Figure 5d). In addition, ARS staining showed that the extent of calcium deposition decreased after transfection with antago miR-29b-3p, and the inhibition of calcium deposition induced by transfection with sh-XIST was reversed (Figure 5e).

** P < 0.001 vs. blank; ## P < 0.001 vs. antagomiR-29b-3p.

### MiR-29b-3p targeted NNMT

**Figure 6.**
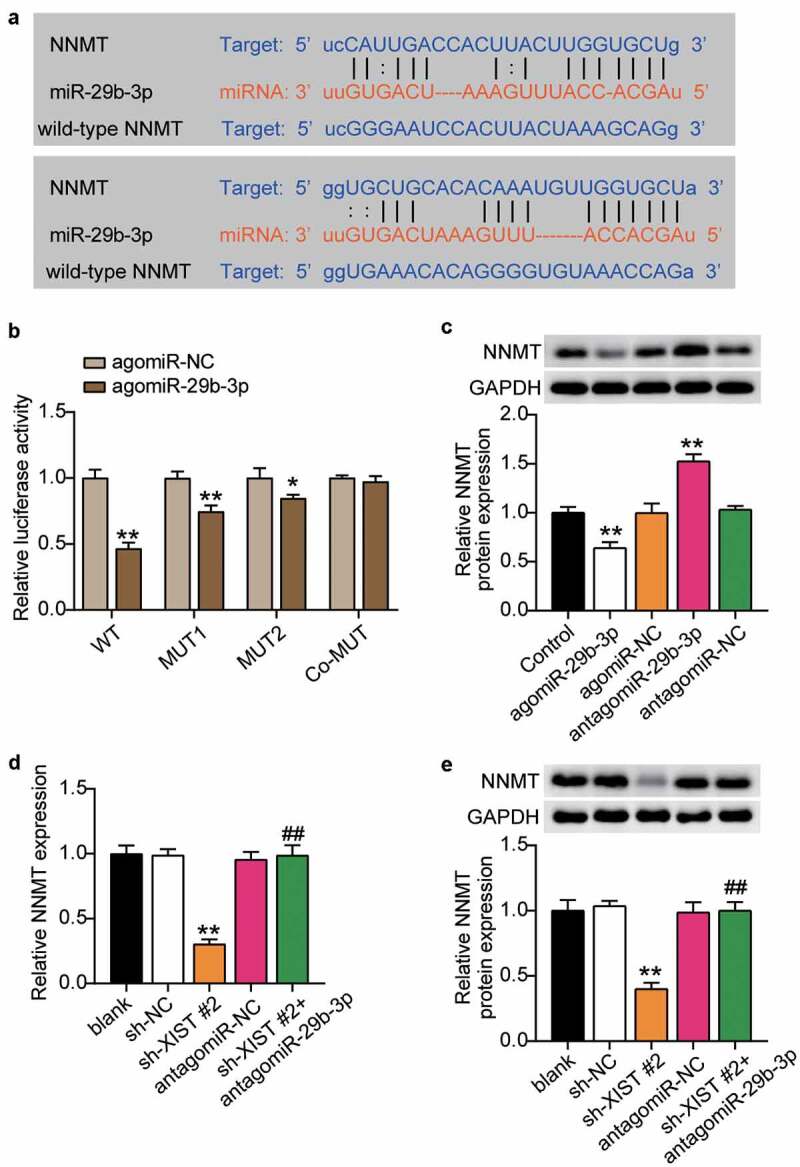
**MiR-29b-3p targeted NNMT**.(a) Schematic diagram of predicted binding sites of NNMT in 3’-UTR of miR-29b-3p. (b) Determination of the dual-luciferase activity of BMSCs transfected with XIST-MUT (MUT1, MUT2 and Co-MUT) or NNMT-WT and agomiR-NC or agomiR miR-29b-3p. * P < 0.05, ** P < 0.001 vs. agomiR-NC. (c) The protein expression of NNMT in BMSCs transfected with antagomiR miR-29b-3p or agomiR miR-29b-3p. ** P < 0.001 vs. NC. (d) The mRNA expression of NNMT in BMSCs after transfection of antagomiR miR-29b-3p or sh-XIST 2#. (e) The protein expression of NNMT in BMSCs after transfection of antagomiR miR-29b-3p or sh-XIST 2#

After confirming that NNMT was related to XIST and miR-29b-3p, we applied starBase to find the target sites of miR-29b-3p and NNMT, and found that miR-29b-3p contained two binding sites for NNMT (Figure 6a). Dual-luciferase reporter assay to detect the binding relationship showed that mutations in both binding sites had no significant impact on the luciferase activity. However, mutation of one site decreased the luciferase activity by about 20%, while when two binding sites were included, luciferase activity decreased by 60% (Figure 6b), indicating that miR-29b-3p targeted the NNMT 3’-UTR. Inhibition of miR-29b-3p upregulated the expression of NNMT, while its overexpression decreased the level of NNMT (Figure 6c), suggesting that miR-29b-3p negatively regulates NNMT by binding to its 3’-UTR. Next, we examined the effect of miR-29b-3p on osteogenic differentiation through NNMT. RT-qPCR and western blots showed that levels of NNMT mRNA and protein decreased after XIST knockdown, while inhibition of miR-29b-3p reversed this effect (Figure 6d, e). These results indicate that XIST positively regulates NNMT expression.

** P < 0.01. ** P < 0.001 vs. blank; ## P < 0.001 vs. sh-XIST #2.

### MiR-29b-3p inhibitor relieved the effect of silencing NNMT on osteogenic differentiation

**Figure 7.**
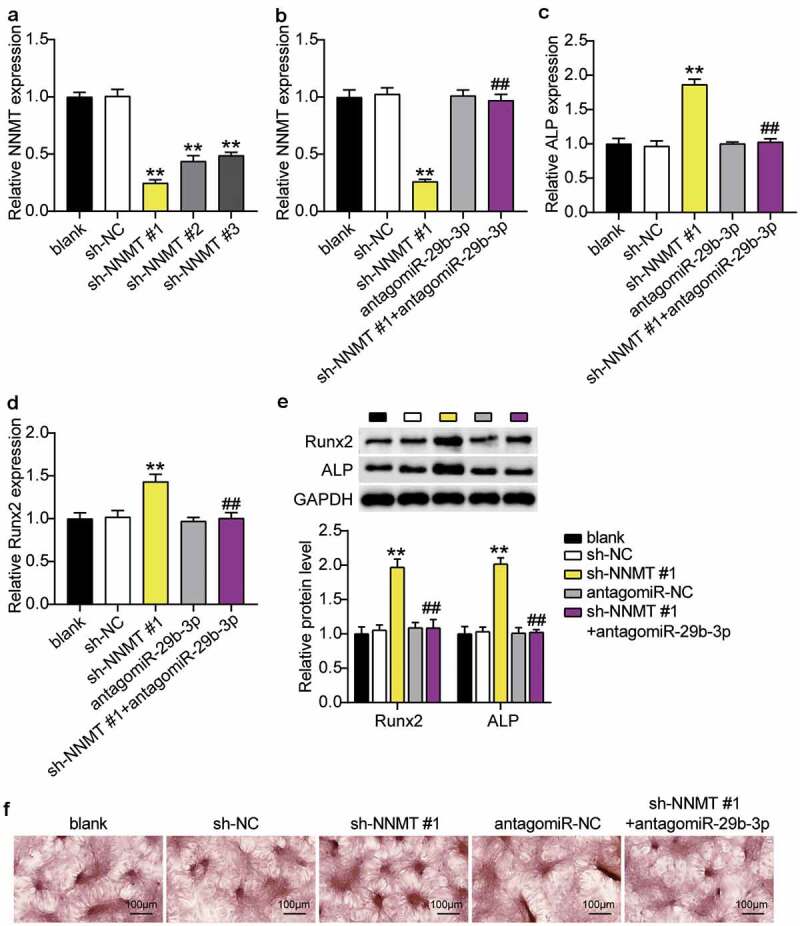
**MiR-29b-3p promotes osteogenic differentiation by inhibiting NNMT**.(a) The NNMT mRNA expression in BMSCs after transfection of sh-NNMT #1, sh-NNMT 2# or sh-NNMT 3#. (b) The NNMT mRNA expression in BMSCs after transfection of sh-NNMT #1 or antagomiR miR-29b-3p. (c) The expression of ALP mRNA in BMSCs after transfection of antagomiR miR-29b-3p or sh-NNMT #1. (d) The expression of RUNX2 mRNA in BMSCs after transfection of antagomiR miR-29b-3p or sh-NNMT #1. (e) The expression of RUNX2 and ALP protein in BMSCs after transfection of antagomiR miR-29b-3p or sh-NNMT #1. (f) Calcium deposition in BMSCs transfected with antagomiR miR-29b-3p or sh-NNMT #1 was detected by ARS staining

When sh-NNMT#1, sh-NNMT#2 or sh-NNMT#3 was transfected into BMSCs, expression level of NNMT decreased, and was the lowest with sh-NNMT #1 (Figure 7a). RT-qPCR showed that the levels of NNMT mRNA decreased after interference with NNMT, which was reversed with miR-29b-3p knockdown of NNMT (Figure 7b). ALP and RUNX2 mRNA in the sh-NNMT #1 group were, respectively, 1.8 and 1.4 times higher than that in the sh-NC group, whereas transfection of antago miR-29b-3p partially countered the effect of sh-NNMT #1(Figure 7c, d). In addition, western blots showed that ALP and RUNX2 increased 2.0-fold and 1.9-fold, respectively, when NNMT was knocked down, and after knocking down miR-29b-3p and NNMT, were similar to the blank group (Figure 7e). In addition, ARS staining showed that inhibition of NNMT expression increased the calcium nodules, while inhibition of miR-29b-3p expression reversed this (figure 7f).

** P < 0.001 vs. blank; ## P < 0.001 vs. sh-NNMT #1.

## Discussion

During normal cycle of bone metabolism, bone formation and resorption are in dynamic equilibrium to optimize bone remodeling [13]. However, disturbance of the coupling balance may lead to enhanced bone resorption by osteoclasts or a decreased bone formation by osteoblasts, leading to OP [17]. At present, most of the drugs available for the treatment of OP still have several shortcomings, including poor safety, and severe side effects, such as increase in the rates of cancer-related morbidity and cardiovascular complications [18]. Therefore, maintaining the osteogenic abilities of BMSCs, thereby reducing the bone loss, is of great clinical value for the prevention and treatment of OP. In the present study, we examined the upregulation of XIST in the serum of the patients of OP, and its downregulation during osteogenic differentiation. Loss and gain of function demonstrated that downregulation of XIST promotes osteogenic differentiation of BMSCs, while the upregulation of XIST led to contradictory effects. Bioinformatic analyses showed that XIST acted as a sponge for miR-29b-3p, and upregulated the NNMT expression to interfere with the osteogenic differentiation, resulting in dysfunction of BMSCs. Our findings revealed a novel mechanism of BMSC dysfunction, suggesting a promising approach for OP intervention.

Previous studies have demonstrated that XIST participates in the OP [14,19,20]. Here, we found that XIST is upregulated in the patients of OP. Moreover, expression level of XIST decreased as osteogenic differentiation of BMSCs progresses. Several studies have shown that ALP is a key marker of early osteogenic differentiation of BMSCs and is important for normal BMSCs [21]. Runx2 is also an important marker of bone formation, and indirectly participates in osteoblast activation during osteogenesis [22]. Calcium nodules are a marker of maturation of BMSCs, and are used to analyze the osteogenic differentiation of BMSCs [23]. Results of our osteogenic differentiation experiments showed that overexpression of XIST lowered the expression of ALP and Runx2 mRNA and proteins, as well as the calcium nodules. Therefore, consistent with the findings of Chen et al. [19], our results suggest that XIST may promote osteogenic differentiation in patients of OP.

MiR-29b-3p is associated with osteogenic differentiation. Zhang et al. [24] reported that the high expression of miR-29b-3p in the BMSCs from the patients of OP, and the osteogenic differentiation of BMSCs was mediated by OP-related genes OPN, OCN, and ALP. The results of Feichtinger et al. [15] showed that miR-29b-3p has a significant correlation with histomorphometric and microstructure parameters of bone formation, which may reflect the dynamics of bone formation and microstructure. In this study, we observed a low expression of miR-29b-3p in patients of OP, and *in vitro* knockdown of miR-29b-3p inhibited ALP and Runx2 expression, as well as formation of calcium nodules. Our results revealed the underlying mechanism of miR-29b-3p action in OP. Bioinformatics and luciferase assays showed that XIST targets miR-29b-3p in BMSCs. In addition, rescue experiments showed that low expression of miR-29b-3p eliminated the effect of interference with XIST on osteogenic differentiation. These results suggest that XIST acts through miR-29b-3p in OP.

NNMT is expressed in many tissues, including the liver, adipose tissue, and skeletal muscle [25]. A recent study showed that NNMT is associated with metabolic diseases and cancer [26]. Agrawal et al. [27] found that NNMT expression was correlated to the transcription factors necessary for osteogenic differentiation of human BMSCs. Wang et al. [12] proved that NNMT was regulated by key circRNAs of BMSCs in ovariectomized mice. In this study, we found that silencing of NNMT inhibited ALP and Runx2 expression in BMSCs, and the extent of calcium nodule formation. Furthermore, it was shown that NNMT, as a target of miR-29b-3p, and NNMT silence can rescue the effect of miR-29b-3p inhibitor on differentiation. These observations suggest that, the miR-29b-3/NNMT axis may be downstream effectors of XIST in OP.

However, this study has some limitations. The status of XIST and miR-29b-3p are screened through GEO DataSets only, their status in other databases, such as TCGA are not yet explored. Lack of animal models is a drawback. In future, we will establish a model of osteoporosis with ovariectomized rats, to study the effect of XIST/miR-29b-3p/NNMT signal axis on OP. In addition, mechanism of downstream control of NNMT will also be the focus of future research.

## Conclusions

In conclusion, as a competitive endogenous RNA of the miR-29b-3p sponge that regulates the expression of NNMT, XIST plays a role in inhibiting osteogenic differentiation in OP. The newly discovered XIST/miR-29b-3p/NNMT axis provides new evidence for the mechanism of osteogenic differentiation of BMSCs, and may offer a theoretical basis for designing new treatments for OP.

## Supplementary Material

Supplemental Material

## Data Availability

The datasets used and/or analyzed during the current study are available from the corresponding author on reasonable request.

## Highlight

1. XIST is significantly upregulated in osteoporosis.
2. XIST regulates the levels of ALP and Runx2 and interferes with calcium nodules in bone marrow mesenchymal stem cells (BMSCs).
3. XIST/miR-29b-3p/ nicotinamide N-methyltransferase (NNMT) axis suppresses the osteogenic differentiation of BMSCs.
