# Supplementary material for: Long non-coding RNA XIST promotes osteoporosis by inhibiting the differentiation of bone marrow mesenchymal stem cell by sponging miR-29b-3p that suppresses nicotinamide N-methyltransferase
Source: Bioengineered. 2021 Sep 5;12(1):6057–69. doi: 10.1080/21655979.2021.1967711 (PMC8806730; doi:10.1080/21655979.2021.1967711)
Supplement: Supplemental Material [file KBIE_A_1967711_SM9158.zip › Supplementary Table 1.docx]

Supplementary Table 1. The differentially expressed genes in osteoporosis by analyzing GSE35959 data series.

| ID | Adjusted P value^*^ | logFC^#^ | Gene symbol | Gene title |
| --- | --- | --- | --- | --- |
| 222439_s_at | 0.0003614 | 4.460088 | THRAP3 | thyroid hormone receptor associated protein 3 |
| 216563_at | 0.007604 | 4.4351058 | ANKRD12 | ankyrin repeat domain 12 |
| 206029_at | 0.0485172 | 3.9932706 | ANKRD1 | ankyrin repeat domain 1 |
| 206373_at | 0.0074827 | 3.9014489 | ZIC1 | Zic family member 1 |
| 216450_x_at | 0.0100178 | 3.8893686 | HSP90B1 | heat shock protein 90 beta family member 1 |
| 230629_s_at | 0.0126404 | 3.8783199 | EP400 | E1A binding protein p400 |
| 231120_x_at | 0.0419529 | 3.877062 | PKIB | protein kinase (cAMP-dependent, catalytic) inhibitor beta |
| 210042_s_at | 0.0004156 | 3.82201 | CTSZ | cathepsin Z |
| 234701_at | 0.0018391 | 3.7810611 | ANKRD11 | ankyrin repeat domain 11 |
| 1553749_at | 0.0409668 | 3.758778 | FAM76B | family with sequence similarity 76 member B |
| 201883_s_at | 0.001369 | 3.6268056 | B4GALT1 | beta-1,4-galactosyltransferase 1 |
| 222556_at | 0.0497239 | 3.5288679 | ALG5 | ALG5, dolichyl-phosphate beta-glucosyltransferase |
| 214090_at | 0.0275153 | 3.5049463 | PAWR | pro-apoptotic WT1 regulator |
| 204506_at | 0.0003614 | 3.4093665 | PPP3R1 | protein phosphatase 3 regulatory subunit B, alpha |
| 216222_s_at | 0.0250422 | 3.3708478 | MYO10 | myosin X |
| 200835_s_at | 0.0019546 | 3.3390566 | MAP4 | microtubule associated protein 4 |
| 232050_at | 0.0408536 | 3.2986571 | FLJ42627 | uncharacterized LOC645644 |
| 214078_at | 0.0139898 | 3.2224361 | PAK3 | p21 (RAC1) activated kinase 3 |
| 235057_at | 0.000817 | 3.1367194 | ITCH | itchy E3 ubiquitin protein ligase |
| 206070_s_at | 0.0251506 | 3.1293016 | EPHA3 | EPH receptor A3 |
| 229566_at | 0.0327416 | 3.1275819 | WFDC21P | WAP four-disulfide core domain 21, pseudogene |
| 1558173_a_at | 0.0218853 | 3.0889583 | LUZP1 | leucine zipper protein 1 |
| 1558050_at | 0.0285266 | 2.9903103 | EIF2B5 | eukaryotic translation initiation factor 2B subunit epsilon |
| 233261_at | 0.0421601 | 2.9821368 | EBF1 | early B-cell factor 1 |
| 224563_at | 0.0254472 | 2.9812059 | WASF2 | WAS protein family member 2 |
| 242660_at | 0.0254106 | 2.9594498 | MALRD1 | MAM and LDL receptor class A domain containing 1 |
| 226675_s_at | 0.0016724 | 2.9550843 | MALAT1 | metastasis associated lung adenocarcinoma transcript 1 (non-protein coding) |
| 1555441_at | 0.0453844 | 2.954329 | UBA6 | ubiquitin like modifier activating enzyme 6 |
| 227702_at | 0.035451 | 2.9376312 | CYP4X1 | cytochrome P450 family 4 subfamily X member 1 |
| 224170_s_at | 0.0086917 | 2.9293953 | TULP4 | tubby like protein 4 |
| 238063_at | 0.0195523 | 2.9179014 | TMEM154 | transmembrane protein 154 |
| 201026_at | 0.0039468 | 2.8926942 | EIF5B | eukaryotic translation initiation factor 5B |
| 1569934_at | 0.0154452 | 2.8899957 | PREX2 | phosphatidylinositol-3,4,5-trisphosphate dependent Rac exchange factor 2 |
| 230200_at | 0.0072213 | 2.8891222 | NSUN6 | NOP2/Sun RNA methyltransferase family member 6 |
| 1561264_at | 0.0450687 | 2.8788766 | LOC105375273 | uncharacterized LOC105375273 |
| 223122_s_at | 0.0226348 | 2.8092308 | SFRP2 | secreted frizzled related protein 2 |
| 1559022_at | 0.0117115 | 2.7535053 | EFCAB14 | EF-hand calcium binding domain 14 |
| 206487_at | 0.0060246 | 2.689513 | SUN1 | Sad1 and UNC84 domain containing 1 |
| 208710_s_at | 0.0362742 | 2.6692071 | AP3D1 | adaptor related protein complex 3 delta 1 subunit |
| 206108_s_at | 0.0375421 | 2.6689196 | SRSF6 | serine and arginine rich splicing factor 6 |
| 231559_at | 0.0073024 | 2.6538727 | NNMT | nicotinamide N-methyltransferase |
| 230885_at | 0.0250827 | 2.6460206 | LOC101930112///SPG7 | uncharacterized LOC101930112///SPG7, paraplegin matrix AAA peptidase subunit |
| 238915_at | 0.0477409 | 2.6324608 | SMIM10L1 | small integral membrane protein 10 like 1 |
| 226220_at | 0.0344233 | 2.6236582 | METTL9 | methyltransferase like 9 |
| 232197_x_at | 0.010496 | 2.5950322 | ARSB | arylsulfatase B |
| 232940_s_at | 0.0024714 | 2.5931571 | KMT2C | lysine methyltransferase 2C |
| 226762_at | 0.0020724 | 2.5898654 | MIR4657///PURB | microRNA 4657///purine rich element binding protein B |
| 202840_at | 0.000817 | 2.5881401 | TAF15 | TATA-box binding protein associated factor 15 |
| 1568680_s_at | 0.0014551 | 2.5803573 | YTHDC2 | YTH domain containing 2 |
| 228581_at | 0.0157296 | 2.5716127 | KCNJ10 | potassium voltage-gated channel subfamily J member 10 |
| 66053_at | 0.0151017 | 2.5639233 | HNRNPUL2-BSCL2///HNRNPUL2 | HNRNPUL2-BSCL2 readthrough (NMD candidate)///heterogeneous nuclear ribonucleoprotein U like 2 |
| 232437_at | 0.0292063 | 2.5317125 | CPSF3L | cleavage and polyadenylation specific factor 3-like |
| 214464_at | 0.0015656 | 2.5281925 | CDC42BPA | CDC42 binding protein kinase alpha |
| 209485_s_at | 0.039864 | 2.5242276 | OSBPL1A | oxysterol binding protein like 1A |
| 202028_s_at | 0.0010904 | 2.5147419 | RPL38 | ribosomal protein L38 |
| 229611_at | 0.0317279 | 2.5090388 | LMLN | leishmanolysin like peptidase |
| 224579_at | 0.0263706 | 2.5059166 | SLC38A1 | solute carrier family 38 member 1 |
| 1569385_s_at | 0.0234232 | 2.4816932 | TET2 | tet methylcytosine dioxygenase 2 |
| 227910_at | 0.0497239 | 2.4747502 | XPNPEP3 | X-prolyl aminopeptidase 3 |
| 205559_s_at | 0.0128112 | 2.4691564 | PCSK5 | proprotein convertase subtilisin/kexin type 5 |
| 201693_s_at | 0.0374205 | 2.4677174 | EGR1 | early growth response 1 |
| 203168_at | 0.0098524 | 2.4673093 | ATF6B | activating transcription factor 6 beta |
| 231768_at | 0.0122646 | 2.4531425 | USF1 | upstream transcription factor 1 |
| 1559427_at | 0.0324793 | 2.4217311 | MCF2L | MCF.2 cell line derived transforming sequence like |
| 213736_at | 0.035627 | 2.420957 | COX5B | cytochrome c oxidase subunit 5B |
| 214953_s_at | 0.0047903 | 2.4042039 | APP | amyloid beta precursor protein |
| 208900_s_at | 0.012783 | 2.3883716 | TOP1 | topoisomerase (DNA) I |
| 1557275_a_at | 0.0211067 | 2.377639 | TLCD2 | TLC domain containing 2 |
| 229648_at | 0.0242028 | 2.377123 | ARHGAP32 | Rho GTPase activating protein 32 |
| 211348_s_at | 0.0423571 | 2.3746613 | CDC14B | cell division cycle 14B |
| 201072_s_at | 0.0327983 | 2.3705631 | SMARCC1 | SWI/SNF related, matrix associated, actin dependent regulator of chromatin subfamily c member 1 |
| 241611_s_at | 0.0401286 | 2.3496469 | FNDC3A | fibronectin type III domain containing 3A |
| 228798_x_at | 0.0348863 | 2.3493981 | LOC100289283 | uncharacterized LOC100289283 |
| 204499_at | 0.0127955 | 2.3396789 | AGTPBP1 | ATP/GTP binding protein 1 |
| 238634_x_at | 0.0448011 | 2.3363394 | LIPJ | lipase family member J |
| 239392_s_at | 0.0453241 | 2.2748316 | POGK | pogo transposable element with KRAB domain |
| 205206_at | 0.0254485 | 2.2468948 | ANOS1 | anosmin 1 |
| 1565823_at | 0.0159323 | 2.2346215 | SEPT7 | septin 7 |
| 229999_at | 0.0403628 | 2.2301239 | LOC101928747///RBMX///SNORD61 | uncharacterized LOC101928747///RNA binding motif protein, X-linked///small nucleolar RNA, C/D box 61 |
| 203184_at | 0.0437252 | 2.2283642 | FBN2 | fibrillin 2 |
| 207631_at | 0.0178669 | 2.2094522 | NBR2 | neighbor of BRCA1 gene 2 (non-protein coding) |
| 222667_s_at | 0.0010904 | 2.2021998 | ASH1L | ASH1 like histone lysine methyltransferase |
| 201055_s_at | 0.0029168 | 2.1917652 | HNRNPA0 | heterogeneous nuclear ribonucleoprotein A0 |
| 212207_at | 0.0236892 | 2.1915551 | MED13L | mediator complex subunit 13 like |
| 213002_at | 0.0025336 | 2.1889905 | MARCKS | myristoylated alanine rich protein kinase C substrate |
| 236557_at | 0.0205346 | 2.1865882 | ZBTB38 | zinc finger and BTB domain containing 38 |
| 221683_s_at | 0.0185573 | 2.18173 | CEP290 | centrosomal protein 290 |
| 212649_at | 0.0081178 | 2.1733691 | DHX29 | DEAH-box helicase 29 |
| 209291_at | 0.0223939 | 2.1688035 | ID4 | inhibitor of DNA binding 4, HLH protein |
| 208478_s_at | 0.0053684 | 2.1678111 | BAX | BCL2 associated X, apoptosis regulator |
| 226643_s_at | 0.0073024 | 2.152842 | NUDCD2 | NudC domain containing 2 |
| 226746_s_at | 0.0125674 | 2.1487679 | UBE4B | ubiquitination factor E4B |
| 213790_at | 0.0074827 | 2.1038444 | ADAM12 | ADAM metallopeptidase domain 12 |
| 214311_at | 0.031911 | 2.1023797 | ZFPL1 | zinc finger protein like 1 |
| 229420_at | 0.0056156 | 2.0784435 | LOC101243545///SNORD4A | uncharacterized LOC101243545///small nucleolar RNA, C/D box 4A |
| 209570_s_at | 0.0418553 | 2.0751691 | NSG1 | neuron specific gene family member 1 |
| 236356_at | 0.0033845 | 2.0686808 | NDUFS1 | NADH:ubiquinone oxidoreductase core subunit S1 |
| 214149_s_at | 0.0017734 | 2.0641402 | ATP6V0E1 | ATPase H+ transporting V0 subunit e1 |
| 228477_at | 0.0017515 | 2.0613715 | ARGLU1 | arginine and glutamate rich 1 |
| 1554411_at | 0.006781 | 2.054906 | CTNNB1 | catenin beta 1 |
| 1554903_at | 0.0124233 | 2.0525275 | FRMD8 | FERM domain containing 8 |
| 226485_at | 0.0480209 | 2.0493119 | LOC101928274///VSIG10 | uncharacterized LOC101928274///V-set and immunoglobulin domain containing 10 |
| 215155_at | 0.0419529 | 2.0318077 | HEXA | hexosaminidase subunit alpha |
| 235846_at | 0.0043788 | 2.0208038 | FSBP///RAD54B | fibrinogen silencer binding protein///RAD54 homolog B (S. cerevisiae) |
| 228397_at | 0.0080592 | 2.0193032 | TUG1 | taurine up-regulated 1 (non-protein coding) |
| 221960_s_at | 0.0236892 | 2.0141934 | RAB2A | RAB2A, member RAS oncogene family |
| 202425_x_at | 0.0020876 | 2.0118154 | PPP3CA | protein phosphatase 3 catalytic subunit alpha |
| 224863_at | 0.0193763 | 2.0074243 | GNAQ | G protein subunit alpha q |
| 236966_at | 0.0418526 | 2.0002688 | ARMC8 | armadillo repeat containing 8 |

Selection criteria: ^*^adjusted P<0.05, ^#^|logFC|>=1.5.
